# Growth signaling autonomy in circulating tumor cells aids metastatic seeding

**DOI:** 10.1093/pnasnexus/pgae014

**Published:** 2024-01-25

**Authors:** Saptarshi Sinha, Alex Farfel, Kathryn E Luker, Barbara A Parker, Kay T Yeung, Gary D Luker, Pradipta Ghosh

**Affiliations:** Department of Cellular and Molecular Medicine, School of Medicine, University of California San Diego, La Jolla, CA 92093, USA; Biointerfaces Institute, University of Michigan, Ann Arbor, MI 48109-2200, USA; Biointerfaces Institute, University of Michigan, Ann Arbor, MI 48109-2200, USA; Moores Cancer Center, University of California San Diego, La Jolla, CA 92093, USA; Department of Medicine, School of Medicine, University of California San Diego, La Jolla, CA 92093, USA; Moores Cancer Center, University of California San Diego, La Jolla, CA 92093, USA; Department of Medicine, School of Medicine, University of California San Diego, La Jolla, CA 92093, USA; Department of Biomedical Engineering, University of Michigan, Ann Arbor, MI 48109-2200, USA; Department of Microbiology and Immunology, University of Michigan, Ann Arbor, MI 48109-2200, USA; Center for Molecular Imaging, Department of Radiology, University of Michigan, Ann Arbor, MI 48109-2200, USA; Department of Cellular and Molecular Medicine, School of Medicine, University of California San Diego, La Jolla, CA 92093, USA; Department of Medicine, School of Medicine, University of California San Diego, La Jolla, CA 92093, USA; Veterans Affairs Medical Center, 3350 La Jolla Village Drive, San Diego, CA 92161, USA

**Keywords:** cellular autonomy, GIV, Girdin, CCDC88A, metastasis

## Abstract

Self-sufficiency (autonomy) in growth signaling, the earliest recognized hallmark of cancer, is fueled by the tumor cell's ability to “secrete-and-sense” growth factors (GFs); this translates into cell survival and proliferation that is self-sustained by autocrine/paracrine secretion. A Golgi-localized circuitry comprised of two GTPase switches has recently been implicated in the orchestration of growth signaling autonomy. Using breast cancer cells that are either endowed or impaired (by gene editing) in their ability to assemble the circuitry for growth signaling autonomy, here we define the transcriptome, proteome, and phenome of such an autonomous state, and unravel its role during cancer progression. We show that autonomy is associated with enhanced molecular programs for stemness, proliferation, and epithelial-mesenchymal plasticity. Autonomy is both necessary and sufficient for anchorage-independent GF-restricted proliferation and resistance to anticancer drugs and is required for metastatic progression. Transcriptomic and proteomic studies show that autonomy is associated, with a surprising degree of specificity, with self-sustained epidermal growth factor receptor (EGFR)/ErbB signaling. Derivation of a gene expression signature for autonomy revealed that growth signaling autonomy is uniquely induced in circulating tumor cells (CTCs), the harshest phase in the life of tumor cells when it is deprived of biologically available epidermal growth factor (EGF). We also show that autonomy in CTCs tracks therapeutic response and prognosticates outcome. These data support a role for growth signaling autonomy in multiple processes essential for the blood-borne dissemination of human breast cancer.

**Significance Statement**

A Golgi-localized molecular circuitry has been recently implicated in the orchestration of secrete-and-sense autocrine/paracrine loops that impart self-sufficiency in growth signaling, a.k.a., growth signaling autonomy. Using a transdisciplinary approach, this work shows that growth signaling autonomy is uniquely induced in tumor cells that are in circulation. Circulating tumor cells (CTCs) represent a brutish and risky phase in the lifetime of tumor cells when they are exposed to the immune system and hemodynamic sheer forces, all in the setting of growth-factor starvation. Cancer cells appear to rely on the autonomy circuit to survive and enhance their fitness to seed metastases. Autonomy generates the kind of “eat-what-you-kill” entrepreneurial spirit that minimizes the risk of CTCs dying on an otherwise risky journey.

## Introduction

Metastatic breast cancer (MBC) remains a fatal disease. While ∼5–10% of patients are diagnosed with MBC at initial diagnosis, ∼20–30% of patients with stages I–III breast cancer will eventually recur with MBC. Hence, understanding which cells “seed” metastases in MBC is of paramount importance to improve the management and outcome of these patients.

Metastasis begins with the intravasation of cancer cells from primary tumors, either as single cells or in clusters, into the systemic circulation. These circulating tumor cells (CTCs) must then extravasate from the blood stream and disseminate to distant tissues, where they either remain dormant or give rise to metastases (1–3). However, for CTCs to “seed” metastases, they must survive despite the loss of anchorage to the matrix, exposure to the immune system, and hemodynamic shear forces. The past two decades have witnessed significant technological leaps to help detect, enumerate, and characterize CTCs (reviewed in Refs. (4, 5)). Numerous correlations have been discovered between CTCs and the ability to initiate metastases: abundance (6), phenotypic properties, e.g. enhanced protein translation (7), proliferation (7, 8), epithelial-mesenchymal plasticity (EMP) (9–11), epithelial-type CTCs (with restricted mesenchymal transition (7, 12)) that assemble junctions and circulate as clusters (13–16), CTC ½ life (17), the presence of platelets (15), and immune cells (such as neutrophils (8)) in those clusters, hypoxia (18), and circadian rhythm (nighttime worse than a day (19)). Despite these insights, mechanisms connecting these diverse phenotypes to drive aggressive features of CTCs remain unknown. While clustering, either homotypic, with each other, or heterotypic with platelets provides explanation for how CTCs protect themselves from shear forces and evade the immune system, little to nothing is known about how they survive the journey in the face of a precipitous drop in growth factors (GFs). For example, the concentration of epidermal growth factor (EGF) within primary tumors and xenografts range from ∼1 to 2 ng/mL (20); however, serum EGF levels are much lower and ranges between ∼13 and 14 pg/mL (21). Most of the detectable EGF in serum is biologically inactive and is associated with platelets (22), which is released during the process of coagulation (23). In fact, EGF is virtually undetectable in plasma that is collected in the presence of inhibitors of coagulation (24).

It is perhaps because of these adversities that the process by which CTCs initiate metastases is highly inefficient (2.7% efficiency, as determined using MBC-patient-derived CTCs in xenotransplantation models (25)), implying that only a fraction of CTCs—the fittest of them all—are endowed with tumorigenic and metastatic functionality. What signaling mechanisms and/or molecular machineries impart or maintain CTC fitness in GF-deprived state remains a hot topic of debate, and objective molecular measurements of the metastatic potential of CTCs remain an unattainable holy grail of precision medicine.

In this study, we report the serendipitous discovery of a distinct CTC phenotype, i.e. growth signaling autonomy that is induced in CTCs, but not in primary tumors or established metastases. Growth signaling autonomy, or self-sufficiency in GF signaling, is the first of the six hallmarks of all cancers to be defined (26), and yet remains one of the least well understood. Many cancer cells synthesize GFs to which they are responsive, creating a positive feedback signaling loop called autocrine stimulation (27). In fact, serum-free cell culture studies squarely implicate autocrine secretion of GFs as key support for intracellular mechanisms that impart autonomy (reviewed in Ref. (28)). Recently, using an integrated system and experimental approach, a molecular circuit has been described which is critical for multiscale feedback control to achieve secretion-coupled autonomy in eukaryotic cells (29). This circuit is comprised of two species of GTPases, monomeric Arf1 and the heterotrimeric Gi, coupled by the multimodular scaffold GIV (i.e. *G*α-*i*nteracting *v*esicle associated protein; aka Girdin; gene *CCDC88A*) within a closed-loop circuit that is localized at the Golgi (29, 30). Coupling is initiated only when cells are subjected to restricted growth-factor conditions. Coupling within such a closed-loop control system generates two emergent properties: (i) dose–response alignment behavior of sensing and secreting GFs; and (ii) multiscale feedback control to achieve secretion-coupled growth and survival signaling (31). Consequently, cells with a coupled circuit are self-sufficient in growth signaling, i.e. autonomous, and can survive and achieve homeostasis in GF-restricted conditions; cells in which the circuit is uncoupled (as in GIV knock-out [KO] cells) are not.

In this work, we provide evidence for the requirement of such autonomy in breast cancer CTCs and reveal the biological implications and translational potential of our observations.

## Results

### Study design

**Fig. 1.**
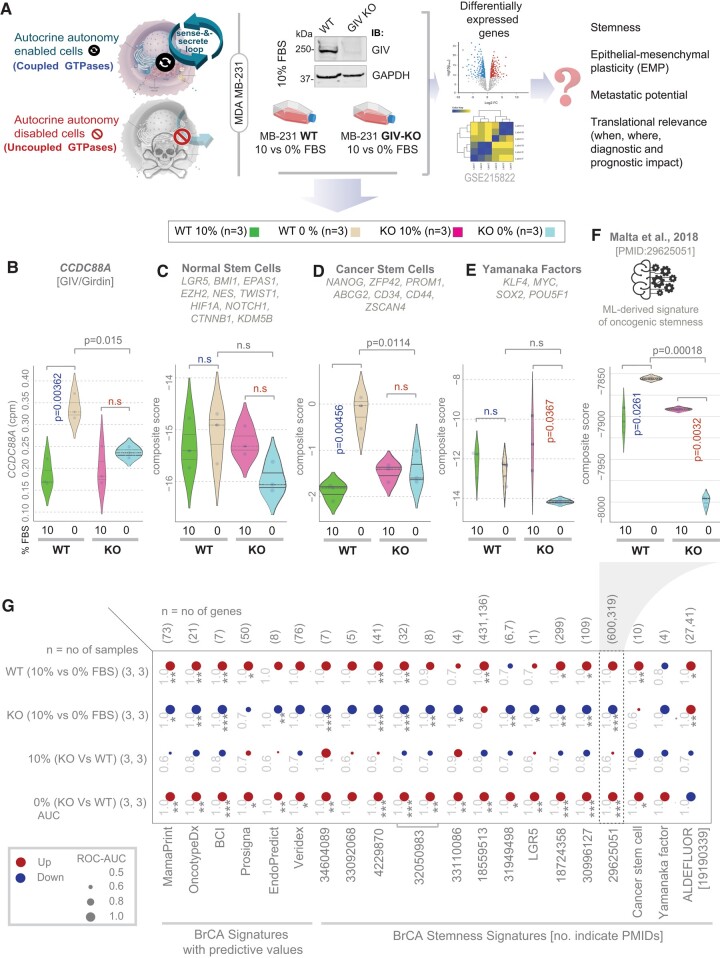
Growth signaling autonomy is required for the induction or maintenance of stemness. A) Schematic displays study design. Autocrine autonomy-endowed cells are compared against cells in which such autonomy is disabled (autonomy impaired) by depletion of GIV (GIV-KO). Cells are grown in the presence or absence of exogenous GFs (0% FBS) to study the biological and translational relevance of autocrine autonomy in breast cancers. B–F) Violin plots display the single (B) or composite (C–F) score of selected gene signatures. *P-*values based on Welch's t test, comparing 10 vs. 0% growth conditions in WT (blue) and KO (red) cells. Blue and red fonts for *P-*values indicate significant up-regulation or down-regulation, respectively. G) Expression of various stemness-associated gene signatures and clinically used breast cancer gene signatures, in parental (WT) vs. GIV-KO (KO, by CRISPR) MDA-MB231 cells grown in 10 or 0% FBS is visualized as bubble plots of ROC–AUC values (radius of circles are based on the ROC–AUC) demonstrating the direction of gene regulation (up, red; down, blue) for the classification of WT and KO samples in 10 and 0% FBS conditions based on the indicated gene signatures (bottom). BCI, breast cancer index. No. indicates PubMed identifier. Statistics: *P-*values based on Welch's t test (of a composite score of gene expression values) are provided either as exact values (in B–F) or using standard code (in panel G; ‘.’*P* <= 0.1, ‘*’*P* <= 0.05, ‘**’*P* <= 0.01,‘***’*P* <= 0.001) next to the ROC–AUC. n.s., not significant. See Fig. S1 for violin plots of representative signatures.

To study how autonomy in cancer cells impacts cancer progression, and more specifically, the progression of breast cancers, we took advantage of two MDA MB-231 [M.D. Anderson Metastatic Breast cancer cell line 231] breast cancer cell lines, that are either endowed (wild-type; WT) or impaired (GIV-KO by CRISPR (29)) in growth signaling autonomy (Fig. 1A). We focused on these cells because they are a highly aggressive, invasive, and poorly differentiated triple-negative breast cancer (TNBC) cell line that lacks the estrogen receptor (ER), progesterone receptor, as well as amplification of the human epidermal growth factor receptor 2 (HER2) and is one of the triple-negative basal subtype cell line most widely used in MBC research (32) (40.2% of total PubMed citations). It is also a cell line that has been shown to require GIV for growth signaling autonomy (29). We analyzed these cells by functional and “omics”-based approaches to navigate the uncharted territory of cancer cell autonomy. Because the GTPase circuit for autonomy requires GIV's modules/motifs that evolved only in the higher eukaryotes (29), and GIV is overexpressed in most cancers (33), we hypothesized that tumor cells may frequently assemble and utilize such an evolutionary advantage to achieve growth signaling autonomy at some stage during cancer progression. Using an integrated computational and experimental approach, we systematically analyzed these pairs of cell lines for key hallmarks of cancer cells.

### Growth signaling autonomy is associated with stemness and EMP

The expression of *CCDC88A* gene (which encodes GIV) was significantly up-regulated when the autonomous WT, but not GIV-KO cells were switched from 10 to 0% serum conditions (Fig. 1B), which is consistent with the increased need for autocrine growth-factor signaling during serum starvation (absence of exogenous GFs, i.e. 0% Fetal bovine serum (FBS)). Although conventional markers of normal pluripotent stem cells remained unchanged during serum depravation in both cells (Fig. 1C), markers of cancer stem cells followed the same pattern as *CCDC88A* (Fig. 1D). When we analyzed the core pluripotency master regulators (the Yamanaka factors (34); Fig. 1E) and the breast cancer-specific indices of the degree of oncogenic dedifferentiation (identified using a machine learning algorithm (35); Fig. 1F), the autonomous WT cells maintained these signatures despite serum depravation; GIV-KO cells did not. These patterns (induction or maintenance in the autonomous WT, but suppression in GIV-KO cells) were observed repeatedly across a comprehensive panel of gene signatures of breast cancer aggressiveness and stemness that have been reported in the literature (Figs. 1G and S1).

**Fig. 2.**
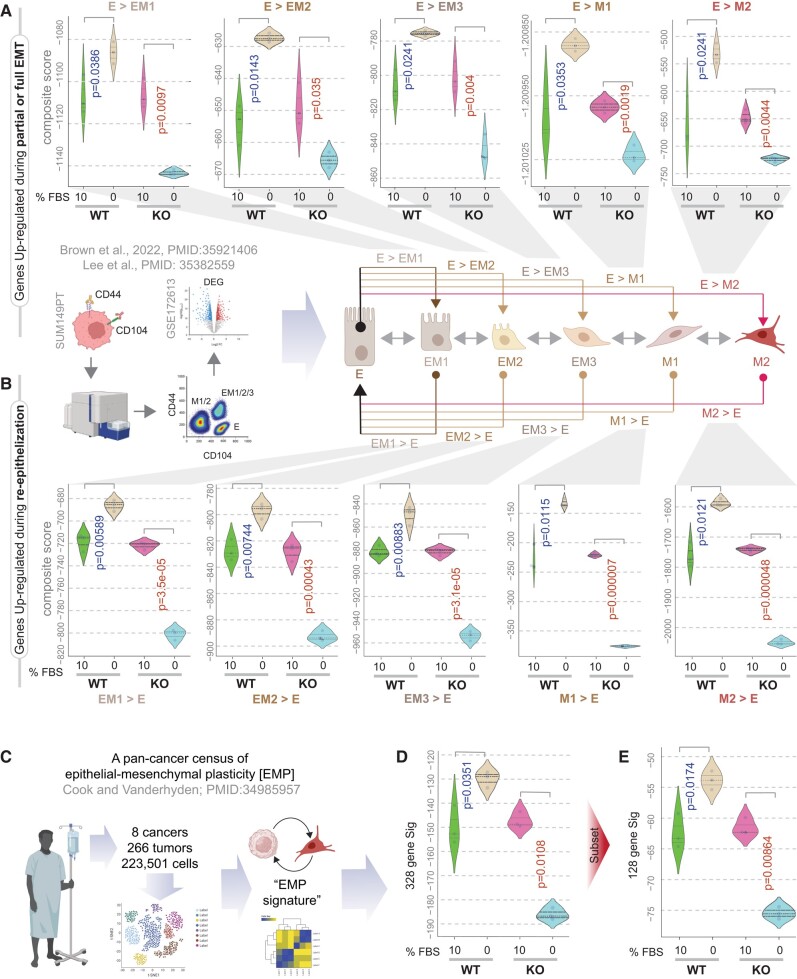
Growth signaling autonomy is required for the induction of molecular programs for EMT, re-epithelization, and EMP. A) Violin plots display the composite score of various published gene signatures (37) in parental (WT) and GIV-depleted (GIV-KO) MDA MB-231 cells, which are induced during epithelial cell transition into partial (EM1, EM2, EM3) or full (M1 and M2) EMT states. B) Violin plots display the composite score of various published gene signatures (38) in parental (WT) and GIV-depleted (GIV-KO) MDA MB-231 cells, which are induced during the re-epithelization of partial (EM1, EM2, EM3) or full (M1 and M2) EMT states. C–E) Schematic (C) summarizes the study design that resulted in the discovery of the pan-cancer transcriptional census of EMP derived by single cell seq (39). Violin plots (D and E) display the composite score of the EMP signature in parental (WT) and GIV-depleted (GIV-KO) MDA MB-231 cells. Panel D shows the expression profile of all 328 genes in the EMP signature, representing various cells in the tumor microenvironment. Panel E shows the expression profile of a subset of 128 genes in the EMP signature that is specifically enriched in cancer cells. *P-*values based on Welch's t test, comparing 10 vs. 0% growth conditions in WT (blue) and KO (red) cells. Blue and red fonts for *P-*values indicate, significant up-regulation or down-regulation, respectively. See Fig. S2 for other gene signatures for EMT, CTC clusters, and MET.

Autonomous WT, but not GIV-KO cells also induced gene signatures for EMP (36), i.e. the ability of cells to interconvert between epithelial and mesenchymal phenotypes in response to signals (Fig. 2). For example, all gene signatures derived from isolated distinct single-cell clones from the SUM149PT human breast cell line spanning the E↔EM1-3↔M1-2 spectrum, previously characterized for diverse migratory, tumor-initiating, and metastatic qualities (37), were induced in the autonomous WT, but suppressed in the GIV-KO cells during serum depravation (Fig. 2A and B). Acquisition of EMP plasticity in MDA-MB-231 cells under conditions of serum deprivation is remarkable since these cells typically show a highly mesenchymal phenotype (40). An identical pattern was seen also for the transcriptional census of EMP in human cancers, which was derived by leveraging single-cell RNA-seq data from 266 tumors spanning eight different cancer types (39) (Fig. 2C). This held true for both the 328-gene EMP consensus signature (Fig. 2D), as well as its cancer cell-specific 128-gene subset (Fig. 2E). Furthermore, numerous gene signatures across the epithelial(E)-mesenchymal(M) transition (EMT) and mesenchymal-epithelial transition (MET) spectrum, derived from diverse human samples representing the stages of metastasis (primary tumors, CTCs, and metastases) and genes that are essential for establishing cell–cell junctions were induced in the autonomous WT, but remained unchanged or were suppressed in the GIV-KO cells (Fig. S2).

These findings indicate that GIV-dependent growth signaling autonomy is required to support molecular programs of stemness and EMP in GF-restricted conditions (i.e. 0% FBS); however, such autonomy is largely dispensable in the presence of excess GFs because all readouts were indistinguishable when WT and KO cells were compared at 10% FBS (Figs. 1 and 2).

### Autonomy is required for anchorage-independent growth and metastatic spread

**Fig. 3.**
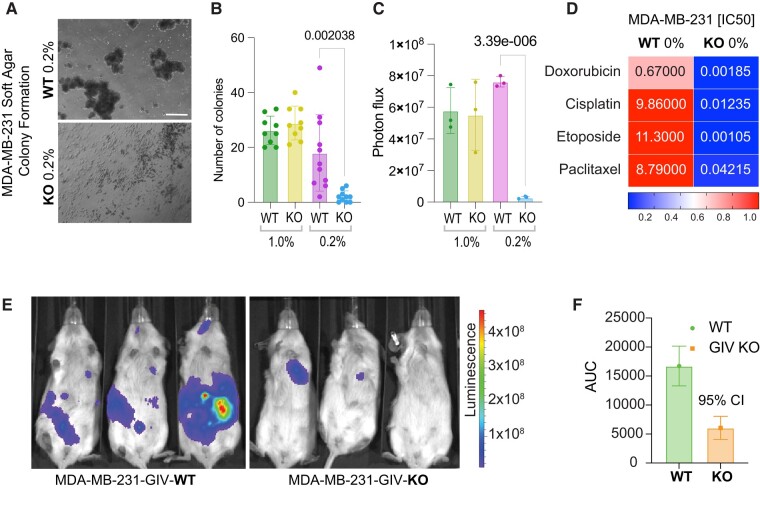
GIV-dependent autonomy is required for growth in GF-restricted conditions, resilience to chemotherapeutics, and metastatic seeding. A–C) Images (A) display representative fields acquired by light microscopy from soft-agar colony growth assays (see the Materials and methods section) on luciferase-expressing parental (WT) and GIV-KO MDA-MB-231 cells, conducted in serum-restricted conditions. Scale bar = 30 µm. Bar plots (B) display the number of colonies/high-power field (HPF) of parental (WT) and GIV-KO MDA-MB-231 cells, quantified using images in 1 and 0.2% FBS (0% FBS for 1 week prevents colony formation altogether). Bar graphs (C) display the average photon flux emitted from colonies in above explained experimental setup. Results in (B and C) are representative of three biological repeats, and 20–40 HPFs were quantified in each repeat. Error bars in C represent SEM. *P-*values were calculated by a two-tailed t test. D) IC_50_ values for three commonly used chemotherapeutic drugs, displayed as a heatmap. E and F) Representative whole mouse bioluminescence images (E) of mice acquired on day 22 after intracardiac injection of serum-deprived parental (WT) and GIV-KO MDA-MB-231 cells in panel A. The logarithmic pseudo-color scale depicts the range of bioluminescence values with red being the highest and blue the lowest. Bar graphs (F) display mean values ± SEM for AUC for total bioluminescence from days 1 to 29 after intracardiac injection for each group. *n* = 5 mice per condition. See also Fig. S3 for similar studies on MCF7 cells (that lack endogenous GIV).

We next assessed if the autonomy-endowed and autonomy-impaired cells are capable of anchorage-independent growth, which is a hallmark of anoikis resistance and the path to further steps in metastasis (41). A prior work (42), using the same cells (MDA MB-231), showed that depletion of GIV before injecting the cells into the mammary fat pad of nude mice reduced distant metastases; the phenotype in mice was attributed to reduced tumor cell invasion/motility with little or no impact on cell proliferation and apoptosis. We prioritized studies that would accomplish two goals: (i) specifically study the role of growth signaling autonomy that is supported by GIV after ensuring that such autonomy is induced by exposing cells to serum-restricted conditions; (ii) maximize our interpretability of readouts by reducing confounding factors such as tumor cell invasion at the primary site, which also requires GIV. When tested for growth as spheroids in soft agar at varying serum concentrations, while both WT and GIV-KO cells did so in the presence of excess serum (10% FBS), only the autonomous WT cells thrived in serum-restricted conditions (0.2% FBS; Fig. 3A–C). Under serum-restricted growth conditions, the autonomous WT, but not the autonomy-impaired GIV-KO cells were also relatively resistant to various classes of conventional chemotherapeutic agents that are typically used to treat TNBCs (e.g. anthracyclines, alkylating agents), as determined by the observed differences in their half-maximal inhibitory concentrations (IC_50_; Fig. 3D). Compared to the KO cells, the autonomous WT cells also displayed significantly higher metastatic potential after intracardiac injection (Fig. 3E and F). These findings indicate that GIV is required for 3D growth, chemoresistance, and metastasis in serum-restricted conditions and that GIV-dependent growth signaling autonomy may be required for these phenotypes.

Previous work has documented the absence of full-length GIV in MCF7 cells, the most widely used ER-positive breast cancer cell line (43.6% of total PubMed citations (32)). These MCF7 cells depend on the growth hormone estrogen to proliferate. We found that restoring GIV expression in these cells using a Tol2-based transposon vector was sufficient to enable estrogen-independent growth, as determined using the ER-antagonist Fulvestrant (Fig. S3A and B) and Tamoxifen (Fig. S3B). GIV was also sufficient for the growth of MCF7 as spheroids in soft agar under estrogen- and serum-restricted conditions (0.2%; Fig. S3C and D). This impact of GIV on cell growth/survival in serum-restricted conditions was limited to GFs and hormones, but not for the CDK4/6 inhibitor, Palbociclib (Fig. S3B), a commonly used therapeutic agent in metastatic ER+ breast cancers, which blocks the cell cycle transition from G1 to S by inhibiting the kinase activity of the Cyclin-dependent kinase (CDK)/cyclin complex. Findings suggest that GIV-dependent growth signaling autonomy may be sufficient for GF and hormone-restricted growth.

### “Autonomy” represents a distinct cell state that is self-sufficient in EGFR/ErbB growth signaling

**Fig. 4.**
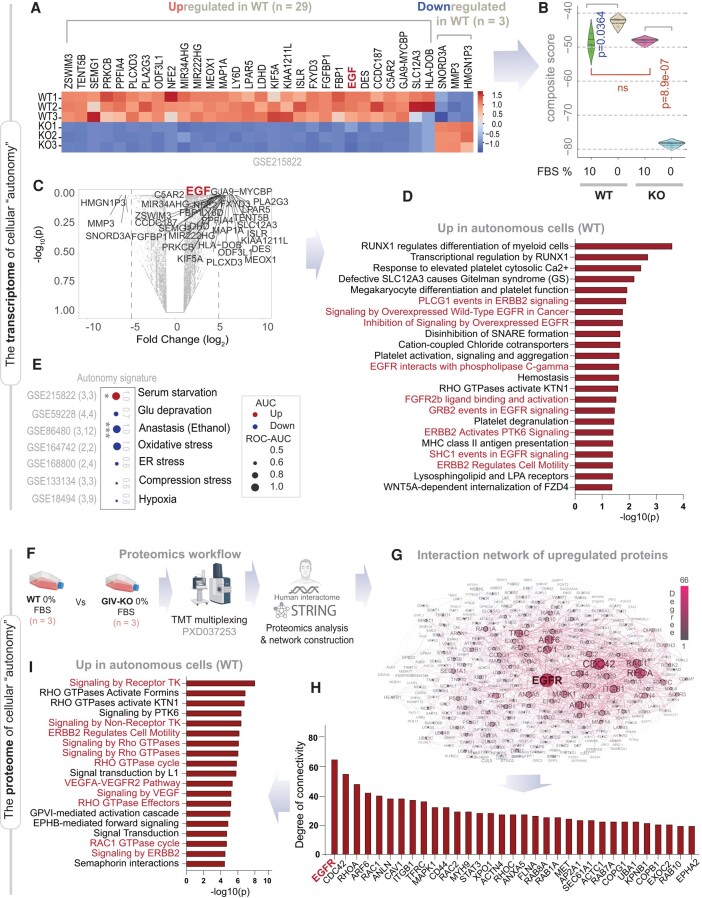
Genes and proteins differentially expressed between autonomy-endowed (WT) and autonomy-impaired (GIV-KO) MDA-MB-231 cells. A) Heatmap displays DEGs (29 up-regulated and 3 down-regulated; LogFC >5, pAdj <0.01) in MDA MB-231 parental (WT) cells compared to its GIV-depleted (KO by CRISPR) counterparts grown in 0% FBS for 16 h. B) Violin plots display the composite score of the DEGs in a (used as an “autonomy signature”) in parental (WT) and GIV-depleted (GIV-KO) MDA MB-231 cells. C) Volcano plot of the differentially expressed genes between WT and GIV-KO MDA MB-321 cells. D) Reactome pathway analyses of the pathways enriched in the genes up-regulated in WT vs. KO MDA MB-231 cells. Red, pathways associated with EGFR/ErbB signaling. See also Fig. S4 for the Reactome pathway for the genes down-regulated in autonomy-endowed (WT) cells. E) Expression of autonomy signature in cells subjected to various stressors is visualized as bubble plots of ROC–AUC values (radius of circles are based on the ROC–AUC) demonstrating the direction of gene regulation (up, red; down, blue) for the classification of control vs stress conditions. MDA MB-231 cells were used in all except anastasis, which was done in HeLa cells. *P-*values based on Welch's t test (of composite score of gene expression values) are provided either as exact values or using standard code (‘.’*P* <= 0.1, ‘*’*P* <= 0.05, ‘**’*P* <= 0.01,‘***’*P* <= 0.001) next to the ROC–AUC. See Fig. S5A–I for visualization of the data as violin plots. F–H) Schematic (F) of the workflow for proteomic analyses. Briefly, differentially expressed proteins (up-regulated in WT, compared to KO; *n* = 3 samples each), as determined by TMT proteomics were used for protein–protein network construction using the human interactome database curated by STRING (search tool for the retrieval of interacting genes/proteins; https://string-db.org/). The resultant network of PPI is displayed in (G). Node size indicates the degree of connectivity within the network. The degree of connectivity of nodes with a *z*-score of degree (*Z*_d_) ≥1 is represented as a bar plot in (H). I) Reactome pathway analyses of the most connected nodes (*Z*_d_ ≥ 3) that are up-regulated in WT vs. KO MDA MB-231 cells. Red, pathways associated with EGFR/ERBB signaling.

RNA sequencing studies revealed a set of 32 genes (29 up-regulated and 3 down-regulated) were most differentially expressed (DEGs; Log-fold-change >5 and adjusted *P*-value of <0.01; Fig. 4A; Supplementary material S3) between the autonomous WT and the GIV-KO cells. Within the DEGs were three up-regulated lncRNA genes, one down-regulated pseudogene, and one down-regulated snoRNA gene. These genes were up-regulated and down-regulated in WT and KO cells, respectively, in response to serum depravation (Fig. 4B), a pattern that was like those we observed previously for signatures of stemness (Fig. 1G) and EMP (Fig. 2A–E). Of the 32 DEGs, eight genes are closely related to various stemness-related pathways (see classification in Supplementary material S2). No genes were significantly differentially expressed between the two cell lines when cultured in 10% serum. The list of up-regulated DEGs was notable for the presence of EGF (Fig. 4C); a reactome pathway analysis confirmed that this list was significantly enriched in genes that participated in the epidermal growth factor receptor (EGFR)/ErbB-signaling pathway (Fig. 4D). Pathway analysis of the down-regulated DEGs was notable for cellular processes related to the extracellular matrix (ECM), e.g. collagen formation, assembly, and degradation, activation of metalloproteinases and degradation of ECM (Fig. S4).

Using the composite score of expression of the 32 genes as a signature of growth signaling autonomy (henceforth, “autonomy signature”), we navigated a wide range of cellular stress response states to assess specificity to growth-factor deprivation (as opposed to nonspecific “stress response”). The autonomy signature induced in MDA MB-231 cells challenged with serum deprivation (Fig. S5A) but not glucose deprivation, oxidative stress, hypoxia, ER-stress, or mechanical compression (Figs. 4E and S5A–G). The signature was suppressed in HeLa cells undergoing anastasis, a process of resurrection in which cancer cells can revive after ethanol-induced apoptosis (Figs. S5H, I and 4E). Furthermore, the autonomy signature had no overlaps with other signatures that are either approved for clinical use by the Food and Drug Administration or in clinical trial for their utility in the management of breast cancers (Fig. S5J). Together, these findings indicate that the gene set of growth signaling autonomy is unique; they represent a distinct “cellular state” that is induced under serum-restricted conditions and requires GIV.

Tandem mass tag (TMT)-based quantitative proteomics studies (Fig. 4F) revealed that autonomous WT cells differential express a distinct set of proteins, which included EGFR (2.308-fold; Supplementary material S4). When we constructed a protein–protein interaction (PPI) network using this list of up-regulated proteins fetched from the human interactome (see network edges in Supplementary material S5), EGFR emerged as the node with highest degree of connectivity (Fig. 4G and H; Supplementary material S6). The most connected proteins (i.e. nodes of the PPI network with *Z*_d_ ≥ 3) that are up-regulated in autonomous WT cells were, once again, found to be significantly enriched in proteins that participate in the EGFR/ErbB2 signaling pathway (Fig. 4I).

Thus, the transcriptomic and proteomic studies agree; both reveal an up-regulated EGFR/ErbB2 signaling pathway in the autonomous WT cells during serum-restricted conditions and confirm the requirement of GIV in such up-regulation. Intriguingly, both *EGF* gene and EGFR protein emerged from these “omics” studies, with a enrichment of its immediate downstream signaling (e.g. signaling via Grb2, PLCγ, CDC42, Rho, and Rac GTPases), and both secretory and endocytic trafficking proteins (e.g. COP1, RABs, SNARE, EXOC1 ARF6, AP2-subunits, CAV1 proteins; Fig. 4H). Findings show that the transcriptome and proteome of growth signaling autonomy support both autocrine/paracrine secretion and signaling within the EGFR/ErbB pathway. They also provide a gene signature for growth signaling autonomy, which is exclusively induced on-demand during scarcity of resources.

### Growth signaling autonomy is induced in CTCs

**Fig. 5.**
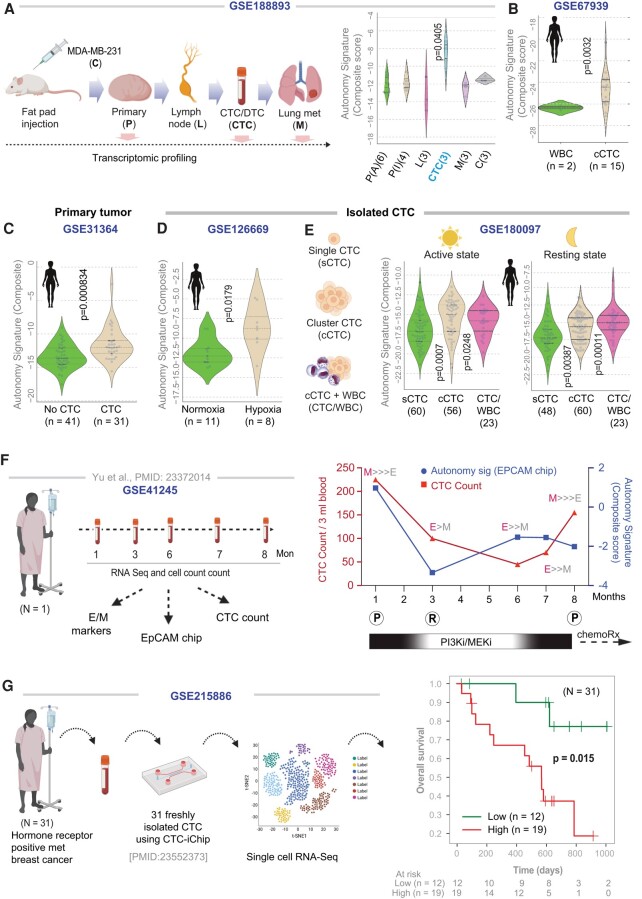
Autonomy signature is induced in CTCs, tracks treatment response, and carries poor prognosis. A) Left: Schematic displays of the study design for a publicly available dataset, GSE103639, in which the MDA MB-231 breast cancer cell line was injected into NOD scid female mice and then RNA-seq was carried out for primary tumors from both axillary (P-A) and inguinal (P-I) locations, lymph nodes (L), isolated CTCs, and metastases to the lung (M). Right: Violin plots display the composite score for autonomy signature in all samples and are assessed for statistically significant differences compared to the primary tumor by Welch's t test. B–E) Violin plots display the composite score for autonomy signature in various human datasets comparing 15 CTC clusters from a single patient during one blood draw against white blood cell (WBC) control (B), primary tumors with/without detectable CTCs (C), CTCs challenged or not with hypoxia (D), and single vs. clustered CTCs collected during different times of the day (E). Statistical significance was assessed by Welch's t test. F) Left: Schematic displays study design used in a publicly available dataset, GSE41245. Right: Graph tracks the abundance of CTCs (CTC count; blue, left *y*-axis), overlaid on composite score of the levels of expression of genes in the autonomy signature in CTCs (as captured using EpCAM-CTC chips, and normalized to counts observed in paired control IgG-chips; red, right *y*-axis). The ratio of epithelial (E) vs. mesenchymal (M) markers, as determined by qPCR and reported in the original study by RTqPCR on a panel of markers is indicated. The *x*-axis (time) is annotated with the therapeutic regimen and clinically determined disease status; (P) progression and (R) response. G) Left: Schematic showing the study design in which CTCs were isolated from 31 unique subjects with MBC using a CTC-iChip microfluidic device and underwent single-cell CTC RNA-seq (GSE215886). Right: KM curves of overall survival on the same cohort, stratified based on high (red) vs. low (green) autonomy signature. Statistical significance (*P-*value) was determined by the log-rank test.

Next, using the newly derived autonomy signature as a computational tool, we sought to navigate the various steps within the cancer initiation and progression cascade. A microarray dataset generated using xenografts of MDA MB-231 cells implanted into inguinal and axillary fat pads of NOD scid female mice, which included samples representing all major steps of the cascade was prioritized (see Fig. 5A, left). To our surprise, the autonomy signature was neither induced in primary tumors nor in metastases; it was induced exclusively in CTC isolated from blood samples (Fig. 5A, right). Findings in mice were conserved in humans; the autonomy signature was induced in human CTCs (Fig. 5B) but not in human primary tumors (when compared to normal, across all molecular subtypes; Fig. S5K). When primary tumors from patients with detectable CTCs were compared to those without, the autonomy signature was higher in tumors that shed CTCs (Fig. 5C). This finding suggests that the features of autonomy are gained in tumor cells before they exit the primary site to become CTCs and that our ability to detect the signature in the CTCs (and not in most primary tumors) could be due to the enrichment of the cells that gain this feature before dissemination into the circulation. Although this degree of specificity (for CTCs) was surprising, the autonomy signature was induced in CTCs is consistent with the near total lack of biologically active EGF in serum (see Introduction).

Next, we interrogated diverse human CTC datasets that were generated by independent groups, in which the metastatic proficiency of the CTCs was experimentally validated using xenograft models. For example, intratumoral hypoxia is a known driver of intravasation of clustered CTCs with high metastatic proficiency (18). We found the autonomy signature significantly higher in hypoxic clusters of live CTCs were compared against their normoxic counterparts (Fig. 5D), all drawn from a breast cancer patient and labeled with HypoxiaRed, a cell-permeable dye that tags hypoxic cells based on their nitroreductase activity (18). The shedding of CTCs is known to peak at the onset of night (43) when they display more metastatic proficiency (19). It is also known that compared to single CTCs, the metastatic proficiency of CTC clusters (15) and CTC-white blood cell (WBC) clusters (8) are higher. The autonomy signature was induced in CTC/CTC-WBC clusters (compared to single CTCs; Fig. 5E); the significance of such induction was higher in CTC-WBC clusters that were collected at night (Fig. 5E).

### Autonomy signature in CTCs tracks therapeutic response and prognosticates outcome

CTCs exhibit dynamic changes in abundance and epithelial and mesenchymal composition during treatment (10); we asked if/how treatment might impact the autonomy signature. We analyzed a dataset comprised of CTCs serially collected during a previously published study on an index patient (Fig. 5F, left), which displayed reversible shifts between these compositions accompanying each cycle of response to therapy (R) and disease progression (P). The autonomy signature was rapidly down-regulated (alongside CTC count) during treatment initiation, which coincided with a therapeutic response (1–3 months; Fig. 5F, right). The signature was subsequently induced from the third to seventh month (despite the continuation of treatment and low CTC counts) and preceded clinically confirmed disease progression at the eighth month which necessitated salvage chemotherapy (Fig. 5F, right). The signature did not show any discernible relationship with the relative amounts of E/M compositions, which is in keeping with our prior observation that autonomy is associated with EM plasticity (Fig. 2).

We next re-analyzed a single-cell RNA-seq dataset generated using 135 viable CTC samples, from 31 unique patients with hormone receptor-positive MBC, for whom follow-up and outcome data were available (overall survival, as updated on 2020 October 2 (7)). CTCs were freshly isolated directly from whole blood using a CTC-iChip microfluidic device (44) (Fig. 5G, left). A Kaplan–Meier (KM) survival analysis revealed a higher 3-year mortality risk among those with high autonomy signature in CTCs compared to those with low expression of the same (*P* = 0.015; Fig. 5G, right).

### Autonomy is associated with the potential to re-epithelialize, evade the immune system, and proliferate

**Fig. 6.**
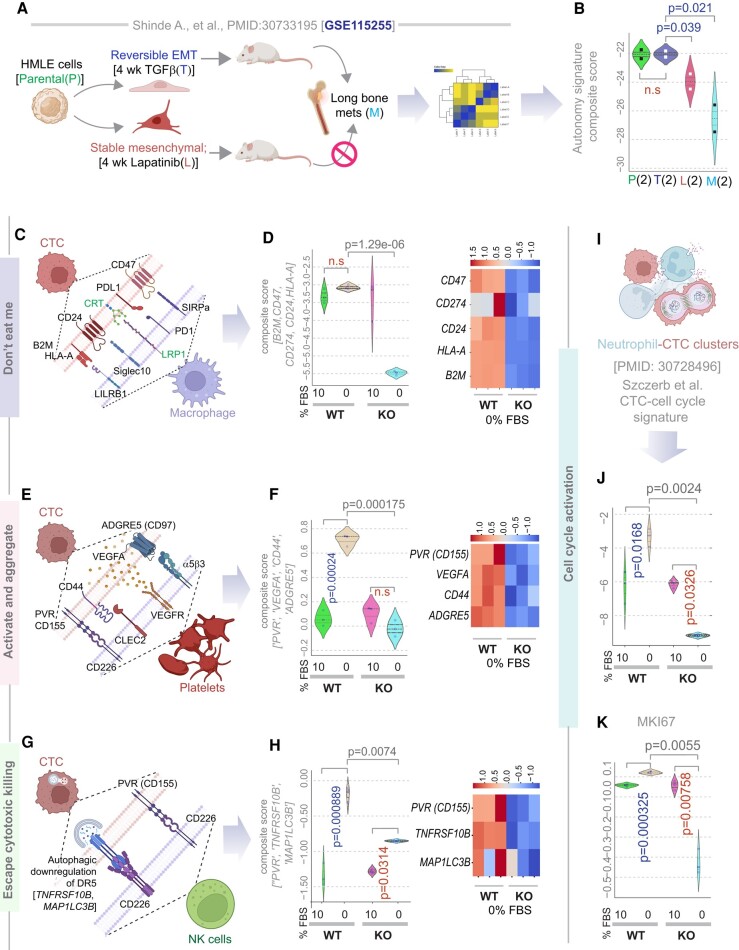
Autonomy is associated with reversible EMT, the capacity to evade immune cells and proliferate. A and B) Schematic (A) displays the key steps in study design using Her2-transformed mammary epithelial cells (HMLE, parental). Induction of reversible EMT (by TGFβ) but not stable EMT (by Lapatinib) is associated with the development of long-distance metastases to bones (BM). Parental, reversible EMT, stable mesenchymal, and bone metastasis-derived clones of HMLE cells were subjected to RNA-seq. Violin plots (B) display the composite score of the genes in the autonomy signature in the HMLE clones. *P-*values based on Welch's t test, comparing TGFβ-induced reversible EMT and other clones. C–H) Schematics summarize the paired CTC-macrophage (C), or CTC-platelets (E), or CTC-natural killer (NK)[cell (G) components known to enhance the metastatic potential of heterotypic CTC clusters (45). Violin plots (left panels; D, F, H) show the composite score of various markers of immune evasion in CTCs in parental (WT) and GIV-depleted (GIV-KO) MDA MB-231 cells. Heatmaps (right panels; D, F, H) show the expression of each gene. See Fig. S6 for violin plots for the individual genes. I–K) Schematic (I) summarizes the study showing cell cycle activation in CTC-neutrophil clusters. Violin plots show either the composite score of expression of genes in that specific CTC-associated cell cycle signature (J) or levels of expression of *MKI67* (K) in parental (WT) and GIV-depleted (GIV-KO) MDA MB-231 cells. Statistics: *P-*values based on Welch's t test. Blue and red fonts for *P-*values indicate, significant up-regulation or down-regulation, respectively.

CTCs must display plasticity between epithelial and mesenchymal states to complete the metastatic process (10), and the EGF/EGFR pathway has been identified as 1 of the 14 major pathways that support EM plasticity (39). We asked if the self-sustained EGF/EGFR signaling program in autonomy is specifically associated with the “reversibility” of the EMT process. We took advantage of a dataset in which Her2-transformed human mammary epithelial (HMLE) cells were either programmed for reversible EMT (induced by Transforming growth factor [TGF]β) or to a stable mesenchymal phenotype (by selection of resistant clones chronically exposed to the ErbB inhibitor, lapatinib; Fig. 6A). Xenograft studies using these programmed cells had confirmed that reversible, but not stable mesenchymal phenotype produces long-bone metastases (46). We found the autonomy signature to be higher in cells programmed for reversible EMT compared to both stable mesenchymal cells and established bone metastases (Fig. 6B). This indicates that the autonomous state is present in transformed cells that carry the potential to undergo dynamic transitions (EM plasticity) but is lost when cells get stuck in either stable mesenchymal (as during the emergence of resistance to Lapatinib) or re-epithelialized states (as in established metastatic colonies). Because Lapatinib-resistance has been attributed in part to the compensatory up-regulation of the autocrine ErbB/EGFR signaling pathways, e.g. heregulin/EGFR (47) and neuregulin1/Her3 (48) signaling circuits, suppressed autonomy signature in lapatinib-treated clones is unlikely to be due to acute suppression of EGF/EGFR signals.

EMT and stemness in tumor cells correlate with immune checkpoint expression and complex interactions with platelet and immune cells (35, 49); similarly, the metastatic proficiency of the CTCs is regulated by interactions with the platelets and immune cells (45, 50). We found that all major CTC markers that are known to be critical for the assembly of the CTC-monocyte (Fig. 6C and D), the CTC-platelet (Figs. 6E, F and S6A–F) and the CTC-natural killer (NK) cell (Fig. 6G and H) synapses were expressed at significantly higher levels in the autonomous WT cells compared to their GIV-KO counterparts exclusively in 0% FBS conditions. These findings suggest that autonomous WT, but not the autonomy-impaired GIV-KO cells are likely to be able to mount an immune evasion (“do not eat me”) response by escaping phagocytosis by monocytes, triggering platelet aggregation and activation which shields CTCs from NK cells, and finally, evading cytolytic killing by NK cells.

Besides immune evasion, CTC-neutrophil interactions are known to induce the expression of CTC genes that outline cell cycle progression, leading to more efficient metastasis formation (45). This neutrophil-related pro-proliferative signature was highly expressed in the autonomous WT cells but suppressed in the autonomy-impaired GIV-KO cells upon serum deprivation (Fig. 6I and J). A similar pattern was seen also for the universal proliferation marker gene, MKI67 (Fig. 6K), and two other gene sets (gene set enrichment analysis) for cell cycle progression, KEGG, and BIOCARTA (Fig. S6G and H).

**Fig. 7.**
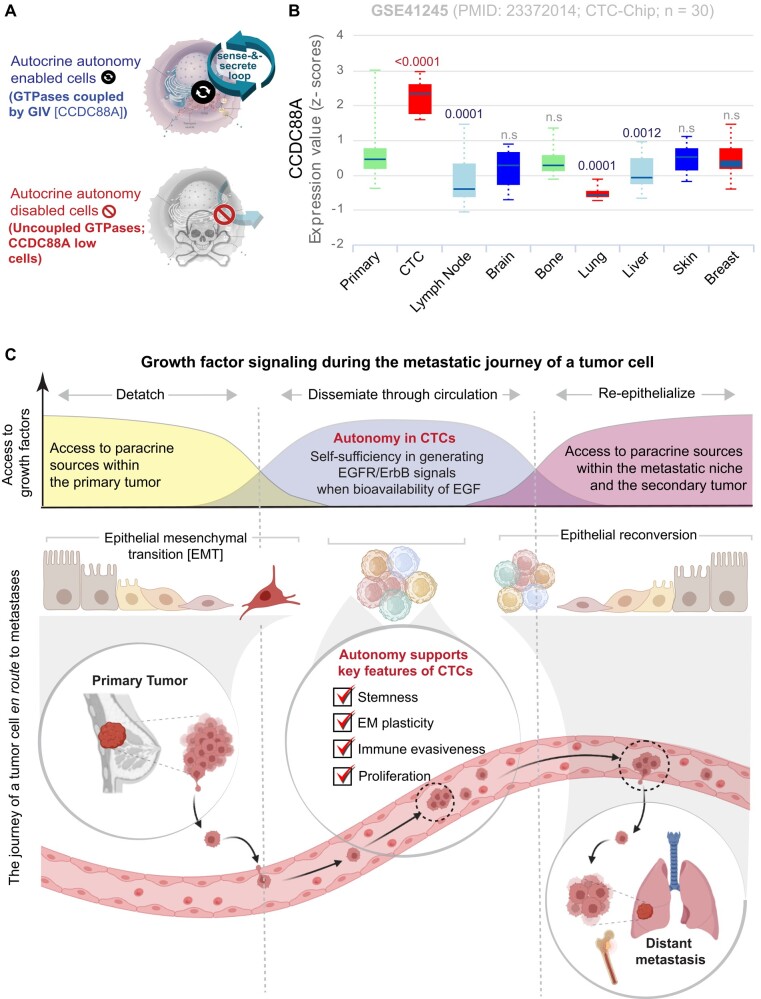
Summary of findings and working model. A) Summary of how the coupling of GTPases by GIV, encoded by *CCDC88A*, gives rise to tumor cells that are empowered (top) or not (bottom) with growth signaling autonomy. B) Box plot showing the *z*-score normalized expression pattern of *CCDC88A* gene in patient samples (GSE41245), as accessed through the publicly available pooled CTC and microemboli portal, ctcRbase. *P*-values are calculated based on paired t test of each type compared to the primary tumor. C) Schematic showing the various phases of the journey of tumor cells enroute to seeding metastases. Top: Growth-factor signaling and source during that journey. Bottom: Epithelial to mesenchymal (EMT) and mesenchymal to epithelial (MET) transition states in primary (left) or metastatic (right) tumors and the transitioning state in CTC clusters. Growth signaling autonomy, which is seen in CTCs, appears to support a self-sufficient EGFR/ErbB-signaling program that is required for re-epithelialization during metastasis. Other properties of autonomous CTCs include stemness, EM plasticity, immune evasiveness, and cell proliferation.

Last, but not least, consistent with the fact that the presence of GIV in our MDA MB-231 cell line models was required for growth signaling autonomy (Fig. 7A), induction of GIV was most prominently seen in patient-derived CTC samples compared to all other steps of metastatic progression (Fig. 7B).

These findings suggest that the autonomous state in the serum-restricted condition is associated with three key CTC properties that are essential for metastasis, i.e. plasticity, immune evasion, and proliferative potential.

## Discussion

In this work, we validate a model system for studying one of the hallmarks of cancers, i.e. self-sufficiency in growth signaling or growth signaling autonomy, define the transcriptome, proteome, and phenome of such autonomous state, and unravel its role during cancer progression. Findings show that the autonomous state is prominently induced in a subset of CTCs before their shedding from the primary tumors and empowers them with key properties that may make them better “seeds” for metastasis (see Fig. 7C). Elaborated below are the three major implications of these findings.

### The autonomous state embodies key features of CTCs that confer metastatic potential

We found that the autonomous state that is unique to a subset of CTCs supports a gene expression program for stemness (i.e. oncogenic dedifferentiation), proliferation, immune evasiveness, and EM plasticity. Of these features, the most distinct one is EMP, because much like what we see in the case of growth signaling autonomy, EMP is unique to CTCs and very rare in primary tumor cells or metastases (51). Autonomy is reduced/lost in cells that are unable to transition between E↔M states. These findings are in keeping with the gathering notion that CTCs are the metastatic precursors that simultaneously express epithelial and mesenchymal markers and best display dynamic E→M and M→E transitions (10). Such a hybrid state in which CTCs have acquired only a partial mesenchymal state allows rapid E→M transitions needed to migrate and intravasate, and quick M→E reversals to reinitiate a tumor at a distant site (52). Consequently, epithelial-type CTCs with a restricted mesenchymal transition initiate metastases efficiently, whereas mesenchymal-type CTCs do not (12). Consistent with prior findings that CTC clusters express cell–cell adhesion proteins that are components of tight junctions and desmosomes (15, 53), we show that the autonomous state is endowed with a gene expression program to support tight junctions (which are essential for the formation of CTC clusters). Divergent from classical EMT, EM plasticity is known to also induce unique immunomodulatory effects (54, 55); consistent with this notion, our model was also accompanied by a diverse array of immune-evasion machinery. Because EM plasticity has been broadly implicated in metastasis, chemoresistance, and immunosuppression (56), and is present in autonomy-endowed cells that outperformed autonomy-impaired cells in their ability to initiate metastases in mice, we conclude that autonomy-endowed CTCs are likely to be more efficient in seeding metastases than those CTCs that are autonomy impaired.

Mechanistically, growth signaling autonomy is supported by a secretion-coupled-sensing circuit at the Golgi apparatus (29, 30) which controls the secretory flux and organelle shape (compact vs. fragmented stacks when the circuit is enabled or disabled, respectively). These findings are in keeping with prior work showing that CTC population-level behavior is dominated by a few high-secreting cells (57), and that Golgi shape (fragmentation vs. compact) is a strong correlate of CTC-EMT and invasiveness (58). Although it remains unknown if/how the Golgi-resident circuit begets EM plasticity, we conclude that growth signaling autonomy and phenotypic plasticity, two hallmarks of cancer, co-exist in CTCs. Because a fraction of CTCs are capable of entering distant sites and persisting as disseminated tumor cells (DTCs) and DTCs are capable of progressing toward metastases or re-entering circulation as CTCs (59), it is intriguing that we did not see a prominent induction of autonomy in DTCs. We believe that could be because most DTCs enter “dormancy” for some period of time and are yet to re-awaken (59–61). While this work was under review, GIV was identified as a key component with a comprehensive catalog of genes and/or proteins “borrowed” by DTCs from bone marrow mesenchymal cells via tunneling nanotubes (in experiments designed to recreate the prolonged and intimate direct contact in the bone marrow niche) (62). It is possible that autonomy can not only be gained before shedding from the primary tumor but also as DTCs in the stromal niches.

### Autonomous CTCs support a self-sufficient EGFR/ErbB-signaling program

Our transcriptomic and proteomic analyses pinpointed with surprising convergence that the autonomous state supports a self-sufficient EGFR/ErbB-centric signaling program in the absence of external GFs. While most cells can be stimulated by GFs made by neighboring cells—via the process of paracrine signaling—many cancer cells acquire self-sufficiency such that they sense and respond to what they synthesize and secrete, creating a positive feedback sense-and-secrete loop known as autocrine stimulation (63). Such a type of self-sufficiency or autonomy in a cancer cell obviates its need to depend exclusively on the surroundings, especially during the intravascular journey (when CTCs cannot access biologically active EGF) and during the initial phase of avascular growth of a CTC that has just extravasated to a new site. Although examples of such autonomy exist in the case of the Platelet-derived growth factor and TGFα by glioblastomas and sarcomas, respectively, and MDA MB-231 cells are known to secrete EGF (64) and depend on its autocrine (not paracrine) sensing/signaling for metastatic progression (65), autocrine autonomy in the EGF/EGFR pathway had not been described previously and mechanisms that support such sense-and-secrete loops in eukaryotes had remained elusive. By demonstrating that a secretion-coupled-sensing machinery at the Golgi (29, 30) that requires scaffolding by GIV is essential for tumor cells to achieve a state of self-sufficiency in EGFR/ErbB signaling, it is not unusual for us to find that such a state is endowed with a multitude of pro-oncogenic biological processes that are known to be supported by EGFR/ErbB signals, including cell anchorage-independent cell growth, stemness, EM plasticity, and metastasis (66). Findings are also in keeping with prior observation of *EGFR* gene induction in EMT that is accompanied by plasticity and tumorigenicity (but not in EMT alone (67)). Although it remains unclear if our findings are related to the previously reported prognostic roles of high EGFR (68, 69) and Her2 (70) in the serum of patients with breast cancers, EGFR/ErbB2 has been detected in CTCs consistently during serial blood draws (71) and was found to be activated (as determined by the presence of its phosphorylated state (72) in CTCs) with increasing frequency often during MBC progression (72). Because inhibition of EGFR prevents CTC clustering and diminishes metastatic potential (73), it is possible that higher autonomy observed in the CTC clusters (compared to single CTCs) requires the autonomous signaling machinery (the sense-and-secrete loop) for CTCs to remain clustered and maintain metastatic potential. It is possible that the EGF-predominant autocrine loop maintains CTC junctions within clusters by triggering both the secretion of junctional proteins/complexes (e.g. E-cadherin) from the Golgi to the plasma membrane (74) and their subsequent activation at the junctions (75). It is equally probable that sheer force in the vascular compartment leads to the mechanical disruption of E-cadherin-EGFR heterotrimeric complexes within CTC clusters, which in turn triggers EGF/EGFR signaling (76), and presumably further augments growth signaling autonomy.

### Autonomy signature could identify CTCs that are the “fittest” precursors to metastases

In the absence of available tools to objectively assess, report, and track the metastatic potential of CTCs, their use in prognosticating the risk of relapse or to track the emergence of therapeutic resistance in real-time and adapt the clinical response has yet to be realized. We show that a gene signature for growth signaling autonomy can track treatment response in a single index patient, and more importantly, the signature prognosticates outcome in a dataset of 31 unique patients. Molecular markers of the metastatic potential of CTCs have been described before, e.g. RPL15 (7) and a 17-gene signature (77). While RPL15 was identified during an in vivo genome-wide CRISPR activation screen to identify genes in breast cancer patient-derived CTCs that promote their distant metastasis in mice, the latter was trained on a cohort of normal vs primary tumor and whole blood from patients. Unlike both these instances, in which investigative approaches were geared to identify CTC-specific markers, we stumbled upon the autonomy signature as a portal into the metastatic proclivity of CTCs by serendipity, using the gene expression signature for autonomy. Regardless, of how it was identified, our findings not only add growth signaling autonomy to the growing list of the parameters that help define the “fitness” of CTCs, but also provide a methodology to objectively measure (using a gene signature) the degree of autonomy (and hence, the fitness) of CTCs to serve as “seeds” for metastases. Prospective studies are required to further investigate the clinical utility of this signature.

In conclusion, our work provides insights into what determines the success of a CTC to serve as a metastatic “seed”. By demonstrating that the detached tumor cells (*sans* ECM contact) gain autocrine self-sufficiency in growth-factor signaling and phenotypic plasticity in circulation while maintaining the properties of stemness, proliferation, and immune evasiveness (which are seen also in primary tumors), we show the coexistence of two hallmarks of cancer that are relatively unique to CTCs and are intricately intertwined.

## Materials and methods

### Experimental methods

#### Cell lines

MDA-MB-231 cells were grown at 37 °C in their suitable media, according to their supplier instructions, supplemented with 10% FBS, 100 U/mL penicillin, 100 μg/mL streptomycin, 1% l-glutamine, and 5% CO_2_. GIV-KO cell lines were generated using pooled guide RNA plasmids (commercially obtained from Santa Cruz Biotechnology; Cat# sc-402236-KO-2), as described earlier (78). Briefly, these CRISPR/Cas9 KO plasmids consist of the green fluorescent protein (GFP) and Girdin-specific 20 nt guide RNA sequences derived from the GeCKO (v2) library and target human Girdin exons 6 and 7. Plasmids were transfected into Hela and MDA-MB-231 cells using polyethylenimine. Cells were sorted into individual wells using a cell sorter based on GFP expression. To identify cell clones harboring mutations in gene coding sequence, genomic DNA was extracted using 50 mM NaOH and boiling at 95 °C for 60 min. After extraction, pH was neutralized by the addition of 10% volume 1.0 M Tris–pH 8.0. The crude genomic extract was then used in PCR reactions with primers flanking the targeted site. Amplicons were analyzed for insertions/deletions (indels) using a Tris/Borate/EDTA–polyacrylamide gel electrophoresis (PAGE) gel. Indel sequence was determined by cloning amplicons into a TOPO-TA cloning vector (Invitrogen) following the manufacturer's protocol. These cell lines were characterized in earlier work (29, 78), and modified here for luciferase expression by transducing cells with a lentiviral vector expressing click beetle green luciferase (CBG) (79). We selected a population of cells stably expressing CBG based on resistance to blasticidin expressed in the lentiviral vector as described previously.

MCF7 cells were purchased from the ATCC and verified cells by short tandem repeat (STR) profiling through the University of Michigan Advanced Genomics Core. We cultured MCF7 cells in Dulbecco's Modified Eagle Medium (DMEM) medium with 10% serum, 1% penicillin/streptomycin, and 1% glutamax (Thermo Fisher Scientific) in an incubator set at 37 °C and 5% CO_2_. We stably expressed CBG in these cells by lentiviral transduction as described previously (79). To stably express full-length, wild-type GIV in MCF7 and MDA-MB-231-GIV KO cells, we used a Tol2 transposon system. The Tol2 transposon vector uses a CAG promoter to drive constitutive expression of the cDNA for *CCDC88A* (the gene name for GIV) and a hygromycin resistance gene linked by a P2A sequence (Vector Builder). We cotransfected cells with the Tol2 GIV transposon and a Tol2 transposase (Vector Builder) at a 3:1 ratio of micrograms of plasmid DNA using Fugene 6 (Promega) according to the manufacturer's directions. Control cells underwent transfection with an empty Tol2 transposon and Tol2 transposase at the same ratio of plasmid DNA. Two days after transfection, we added hygromycin (Sigma-Aldrich) to select batch populations of cells stably expressing GIV. After obtaining stable cell lines, we did not culture resultant MCF7-GIV cells in hygromycin. We stably expressed CBG in MCF7 cells as detailed for MDA-MB-231 cells.

#### Immunoblotting

To verify the expression of GIV in MCF7-GIV and MDA-MB-231-GIV KO/WT cells, equal aliquots of whole-cell lysates, prepared using a Radioimmunoprecipitation assay buffer, were loaded on an 8% Sodium dodecyl-sulfate (SDS)-PAGE gel and immunoblotting was carried out for GIV with the mouse monoclonal antibody H-6 (Santa Cruz Biotechnology) as described previously (80). Immunoblots were analyzed using a Gel Doc system (BioRad, Hercules, CA, USA).

#### RNA sequencing and identification of DEGs

WT and GIV-KO MDA-MB231 cells were grown in 0 and 10% serum concentration in p10 dishes (Corning) for 16 h before harvest, and cell pellets were subsequently processed for RNA extraction using a kit (R2052, Zymo Research) as per manufacturer's protocol. Isolated RNA has been processed for RNA sequencing in the Illumina NovaSeq 6000 platform. Fastq sequence files have been mapped using the human GRCh38 genome. Log-normalized counts per million expression files are submitted to GSE215822. A list of DEGs is provided in Supplementary material S3.

#### TMT proteomics

WT and GIV-KO MDA-MB231 cells were maintained in 0 and 10% serum concentration in p10 dishes (Corning) for 16 h before harvest, and cell pellets were subsequently processed for TMT proteomics using LUMOS Orbitrap-Fusion analyzer. Peptides are identified and mapped using Peaks X Pro pipeline. Intensity ratio of each identified protein in WT MDA-MB231 Vs GIV-KO MDA-MB231 cells has been identified and selected if the significance score >20. The raw proteomics data has been submitted to ProteomeXchange (PXD037253). A list of differentially expressed proteins is provided in Supplementary material S4.

#### Soft-agar growth assays

We performed assays with minor modifications from a published protocol (81). Briefly, we made a base layer of 0.7% agar (Sigma-Aldrich) dissolved in DMEM medium, adding 0.2% serum after the agar solution cooled to ∼37 °C before transferring 1 mL per well to 6-well plates. After the base layer solidified at room temperature, we prepared 0.35% low melting agar (Sigma) in DMEM medium, adding the same concentration of serum as in the base layer and 5 × 10^4^ cells per mL when the solution cooled to ∼37 °C (*n* = 3 wells per cell type and serum condition). We immediately transferred 1 mL per well of the low melting point agar/cell solution to each well and cooled the plate briefly at 4 °C before placing it in a cell culture incubator. We added 0.5 mL fresh DMEM medium with 0.2% serum every 2 days. After one week, we obtained nine bright-field images per well on an inverted microscope (Olympus IX73 with 20× objective). Immediately after microscopy, we added 150 µg/mL luciferin (Promega) to each well; incubated in a cell culture incubator for 10 min; and then acquired a bioluminescence image of total viable cells on an IVIS Lumina (30 s image, large field of view) (Perkin Elmer). A person blinded to experimental conditions enumerated colonies and quantified imaging data (Living Image, Perkin Elmer).

#### Cytotoxicity assays

We performed cytotoxicity assays on MCF7 cells as described previously (82). Briefly, we seeded 7.5 × 10^3^ MCF7 WT or MCF7-GIV cells per well in black wall 96 well plates (Thermo Fisher Scientific, catalog number 165305). One day after seeding in normal growth medium, we washed cells once with Phosphate-buffered saline and then added various concentrations of drugs (tamoxifen, fulvenstrant, or albociclib; all purchased from Tocris) in phenol red free DMEM (Thermo Fisher Scientific) with 4 mM glucose, 1% serum, 1% penicillin/streptomycin, 1% Glutamax, and 10 nM estrogen (*n* = 4 wells per cell type and concentration). Three days later, we added 150 µg/mL luciferin per well; incubated in a cell culture incubator for 10 min; and then acquired a bioluminescence image of total viable cells on an IVIS Lumina (1 min image and large field of view). We quantified bioluminescence as radiance per well (LivingImage software) and normalized data for each cell type and drug concentration to vehicle only.

#### Animal studies

The University of Michigan Institutional Care and Use of Animals Committee approved all animal procedures. We used 8- to 10-week-old female NOD-scid IL2Rgamma^null^ mice originally purchased from The Jackson Laboratory and bred in the colony maintained by the University of Michigan Lab Animal Medicine Program. Before mouse experiments, we cultured MDA-MB-231 WT and MDA-MB-231-GIV KO/WT cells in serum-free DMEM with 25 mM glucose overnight. We verified that these cells did not lose viability after overnight culture in a serum-free medium relative to a medium with 10% serum as determined by cell-based measurements of CBG bioluminescence in an IVIS Lumina (Perkin Elmer). We injected 1 × 10^5^ breast cancer cells per mouse (*n* = 5 per each cell type), verifying the positioning of the 30 g needle in the left ventricle by the return of pulsatile bright red blood as described (83). We imaged bioluminescence with an IVIS spectrum (Perkin Elmer) in mice at time points shown in the figure legend and quantified data with Living Image software. For each mouse, we calculated the fold-change in bioluminescence relative to the value obtained one day after injection to normalize for variations in injected amounts of cells. We calculated area-under-the-curve (AUC) ± SEM for total bioluminescence in each group.

## Computational methods

### Transcriptomic datasets

All publicly available transcriptomic datasets were downloaded from National Center for Biotechnology Information (NCBI) Gene Expression Omnibus website (GEO) (84–86) or European Molecular Biology Laboratory (EMBL) European Bioinformatics Institute (EMBL-EBI) ArrayExpress website (87). All gene expression datasets (Supplementary material S1) were processed separately using the Hegemon (hierarchical exploration of gene expression microarrays online) data analysis framework (88–90). We did not combine datasets that belong to two different platforms. See Supplementary material S1 for the degree of heterogeneity among samples in the datasets used in this work.

### *StepMiner* analysis

*StepMiner* is an algorithm that identifies stepwise transitions using the step function in a time-series data (91). *StepMiner* undergoes an adaptive regression scheme to verify the best possible up and down steps based on sum-of-square errors. The steps are placed between time points at the sharpest change between expression levels, which gives us the information about timing of the gene expression-switching event. To fit a step function, the algorithm evaluates all possible steps for each position and computes the average of the values on both sides of a step for the constant segments. An adaptive regression scheme is used that chooses the step positions that minimize the square error with the fitted data. Finally, a regression test statistic is computed as follows:


$$F\;\mathrm{stat}=\;\frac{\sum_{i=1}^{n}\;{\left(\hat{X_{i}}\;\text{–}\;\bar{X}\right)}^{2}/(m-1)}{\sum_{i=1}^{n}\;{\left(X_{i}-\;\hat{X_{i}}\right)}^{2}/(n-m)}\;$$

where $X_{i}$ for i=1 to n are the values, $\hat{X_{i}}$ for i=1 to n are fitted values. *M* is the degrees of freedom used for the adaptive regression analysis. $\bar{X}$ is the average of all the values: $\bar{X}=\;\frac{1}{n}\times \sum_{\;j=1}^{n}X_{j}.\;\;$ For a step position at *k*, the fitted values $\hat{X_{l}}$ are computed by using $\frac{1}{k}\times \sum_{\;j=1}^{n}X_{j}$ for i=1 to k and $\frac{1}{\left(n-k\right)}\times \sum_{\;j=k+1}^{n}X_{j}$ for i=k+1 to n.

### Composite gene signature analysis using Boolean network explorer

Boolean network explorer (BoNE) provides an integrated platform for the construction, visualization, and querying of a gene expression signature underlying a disease or a biological process in three steps: First, the expression levels of all genes in these datasets were converted to binary values (high or low) using the StepMiner algorithm. Second, Gene expression values were normalized according to a modified *Z*-score approach centered around *StepMiner* threshold (formula = (expr − SThr)/3 × stddev). Third, the normalized expression values for every gene were added together to create the final score for the gene signature. The samples were ordered based on the final signature score. Classification of sample categories using this ordering is measured by ROC–AUC (receiver operating characteristics area-under-the-curve) values. Welch's two sample t test (unpaired, unequal variance [equal_var = False], and unequal sample size) parameters were used to compare the differential signature score in different sample categories. Violin, swarm, and bubble plots are created using python seaborn package version 0.10.1. Pathway enrichment analyses for genes were carried out via the reactome database and algorithm (92). Violin, swarm, and bubble plots are created using python seaborn package version 0.10.1. A list of all gene signatures used in this work is provided in Supplementary material S2.

### Survival outcome analyses

KM analyses were done for different gene signatures. The high and low groups were separated based on *StepMiner* threshold on the composite score of the gene expression values. The statistical significance of KM plots was assessed by log-rank test. KM analyses were performed using lifelines python package version 0.14.6.

### Protein–protein interaction network construction and analysis

Up-regulated proteins in WT MDA-MB-231 cells in comparison with the GIV-KO MDA-MB-231 cells are identified with an intensity ratio cutoff of ≥2 and with a significance value ≥20. Interaction edges between the identified proteins are fetched from the STRING human interactome database and represented in Fig. 4G as a PPI network (PPIN) using Gephi 9.02. The degree distribution of the PPIN is computed using the python network package.

### Statistical analysis

Gene signature is used to classify sample categories and the performance of the multiclass classification is measured by ROC–AUC values. A color-coded bar plot is combined with a density plot to visualize the gene signature-based classification. All statistical tests were performed using R version 3.2.3 (2015 December 10). Standard t tests were performed using python scipy.stats.ttest_ind package (version 0.19.0) with Welch's two sample t test (unpaired, unequal variance [equal_var = False], and unequal sample size) parameters. Multiple hypothesis correction was performed by adjusting *P-*values with statsmodels.stats.multitest.multipletests (fdr_bh: Benjamini/Hochberg principles). Sample number of each analysis is provided with associated plots beside each GSE ID no. or sample name. The statistical significance of KM plots was assessed by log-rank test. Pathway enrichment analyses of gene lists were carried out using the Reactome database (93) (http://reactome.org) and the Cytoscape plug-in, CluGo (http://www.ici.upmc.fr/cluego/cluegoDownload.shtml).

## Supplementary Material

pgae014_Supplementary_Data

## Data Availability

The authors confirm that the data supporting the findings of this study are available within the article and/or its Supplementary material. RNA sequencing and Proteomics data are available in Gene Expression Omnibus (GSE215822, GSE215886) and ProteomeXchange (PXD037253) database.

## Preprints

This manuscript was posted on a preprint: https://doi.org/10.1101/2022.12.02.518910
